# Relationship Between Regular Exercise and Quality of Life Among Middle-Aged and Older Adults in Japan

**DOI:** 10.3390/bs15070978

**Published:** 2025-07-18

**Authors:** Dongshou Yu, Masako Shimura, Masashi Kawanishi

**Affiliations:** 1 Department of Physical Education, Donghua University, Shanghai 201620, China; 2 College of Physical Education, Sports Lifestyle Management, National Institute of Fitness and Sports in Kanoya, Kagoshima 891-2393, Japan; shimura@nifs-k.ac.jp; 3 Long-Life Sports Center, National Institute of Fitness and Sports in Kanoya, Kagoshima 891-2393, Japan; kawanishi@nifs-k.ac.jp

**Keywords:** middle-aged and older adults, exercise habits, exercise frequency, average walking time per session, QoL

## Abstract

This paper clarified the correlation among quality of life (QoL) indicators, exercise implementation level, and exercise habits in middle-aged and older adults under identical exercise intervention conditions. The survey items were anthropometric and physiologic measurements, physical strength measurements, and exercise habits. During the 3-month experimental period, a weekly “health exercise course” served as the primary intervention. For all participants, anthropometric and physiologic measurements, physical strength tests, questionnaire surveys, and other surveys were conducted before and after the experiment; then, the pre- and post-intervention effects were compared. After the exercise intervention, significant differences were observed among middle-aged and older adults in terms of various parameters, such as weight, fat rate, diastolic pressure, systolic pressure, sit-up, standing on one foot, lower limb extension force, activity of daily living (ADL), and subjective well-being (PGC). ADL and PGC changed significantly in the participants who engaged in exercise more than twice a week. However, the participants who engaged in exercise for less than twice a week showed no significant differences in any parameters except the life satisfaction (LSI) mean; the LSI increased in the “Less than twice a week” exercise group but decreased in the “More than twice a week” group. In terms of average walking time per session, the “More than 30 min” exercise group showed significant differences in ADL and PGC, whereas the “Less than 30 min” group showed significant differences only in the LSI. The influence of exercise on QoL indicators of middle-aged and older adults, under the same exercise intervention conditions, is related to their exercise habits. This study highlights the benefits of physical exercise in middle-aged and older adults, emphasizing the importance of regular and sustained exercise for this population. Furthermore, the study provides a scientific basis for improving QoL in middle-aged and older adults, thus, to some extent, addressing the concerns related to the growing population of older adults.

## 1. Introduction

According to the 2024 “National Situation Survey” conducted by the Japanese Ministry of Internal Affairs and Communications, approximately 36.25 million people in Japan are aged 65 years and above, exemplifying a large older adult population in the country. Moreover, the population of older adults accounts for 29.3% of Japan’s total population as of 2023. With the average life expectancy of 81.09 and 87.14 years for Japanese men and women, respectively, Japan ranks first in life expectancy globally. By 2040, the proportion of older adults in Japan is predicted to reach 35.5% of the total population. The number of men and women aged 65 years and above in the country is 15.81 million and 20.55 million, respectively. Additionally, the number of older adults aged over 80, 90, and 100 years has reached 13.19 million, 2.49 million, and 80,000, respectively (Toyosato et al., 2016).

Japan, having entered an “aged society” (Yasunaga et al., 2002) early on and facing an increasingly severe aging situation, constantly grapples with a multitude of social challenges, including “how to alleviate depression and anxiety among older adults” (Brehm, 1999), “how most older adults living alone can take care of themselves in their later years” (Yao et al., 2024), and “how to improve QoL of older adults” (Sato et al., 2009). Despite this, the relationship between exercise interventions and health in older adults remains understudied, particularly in Asia (Yu, 2020).

For older adults, maintaining sufficient physical strength is essential to survive and live independently (Cao et al., 2012). Regular exercise has become a fundamental part of lifestyle and significantly contributes to the improvement of QoL. It has emerged as a key component of national fitness initiatives in the 21st century. Each stage of life presents unique challenges, and in old age, addressing issues such as “physical function”, “retirement from social roles,” “economic factors,” “employment,” and “stress” becomes crucial (Matsumura et al., 2021).

Numerous studies have examined the correlation among exercise habits, physical effects, and QoL, in health promotion projects targeting middle-aged and older adults through exercise interventions. In a swimming intervention study by Hogan and Santomier (1984) on older adults, 78% of the participants reported that they experienced an increased sense of capability not only in swimming but also in various aspects of their daily lives. Sonstroem and Morgan (1989) reported that consistent and regular exercise enhanced the muscle strength and endurance of participants and enabled them to complete daily tasks more effectively in addition to enhancing their self-esteem. Bozoian and McAuley (1994) developed a comprehensive quality of life (QoL) program aimed at fostering sustainable exercise habits.

In recent years, extensive research has been conducted on sports for middle-aged and older adults in Asia, and most studies have focused on aspects such as middle-aged and older adults’ care policies, sports and physical fitness, self-care ability, and psychology (Mai et al., 2021).

However, limited studies have investigated the relationship between QoL and exercise habits, particularly the implementation levels of regular exercise interventions for middle-aged and older adults in Asia.

In this context, the present study explored the effect of and the interrelationship between the exercise habits, specifically the exercise frequency and average walking time per session of an exercise intervention among middle-aged and older adults, and the association of these variables with QoL under standard exercise intervention conditions (Irigaray et al., 2011).

This study provides a scientific basis for optimizing the exercise methods and their intensity in the middle-aged and older adults population, highlighting the importance of routine physical exercise in this population (Villareal et al., 2011).

It also offers a basis for developing practical and suitable exercise goals and plans tailored to individuals’ circumstances.

The main objective of this study is to clarify the correlation among QoL indicators, exercise implementation level, and exercise habits among middle-aged and older adults under identical exercise intervention conditions.

## 2. Materials and Methods

### 2.1. Survey Subjects

This study spanned approximately three months from March to May 2024. A total of 160 healthy individuals aged 60 years and above, without any specific illnesses, were selected as participants (Purath et al., 2009).

The selected participants had earlier participated in the “Health Exercise Promotion Classroom” project in Kanoya City, Kagoshima Prefecture, Japan. Of the total, 17 participants withdrew because of personal reasons, and 11 were excluded because their final data statistics (such as physical strength and questionnaire responses) were deemed invalid.

Finally, 132 participants, comprising 49 men and 83 women, were selected (Table 1). Eight of the participants were aged ≥80 years, and 46 were aged ≥70 years (Table 2). The control group consisted of 73 participants (24 men, 49 women; 3 aged ≥80 years, 26 aged ≥70 years).

### 2.2. Investigation Methods

During the 3-month experimental period, a weekly “health exercise course” served as the primary intervention (Leiper & Craik, 1991). For all participants, anthropometric and physiologic measurements, physical strength tests, questionnaire survey, and other surveys were conducted before and after the intervention (Tsutsumi et al., 1997).

Subsequently, the effects of the exercise intervention were comparatively analyzed (Dayal et al., 2023). Written informed consents were obtained from the participants regarding publication of their clinical data.

All experiments carried out in this study have been approved by the Ethics Committee of Donghua University, China and National Institute of Fitness and Sports in Kanoya, Japan (No.SRSY202412120058, No.32522024NF007). We certify that this study was performed in accordance with the 1964 declaration of Helsinki and later amendments.

### 2.3. Investigation Projects

Survey items considered in this study were anthropometric and physiologic measurements (height, weight, body fat, blood pressure, etc.) (Himann et al., 1988), physical strength measurements (grip strength, sit-and-reach, sit-ups, single-leg standing, lower limb extension) (Rikli & Busch, 1986), and exercise habits (exercise implementation in the past year, including exercise frequency and average walking time per session) (Peig-Chiello et al., 1998) (Table 3).

To assess grip strength, the DM-100S grip tester, manufactured by YAGAMI, was utilized; two tests were conducted for each hand, totaling four tests. In addition, Anaero press500, produced by COMBI, was employed to assess lower limb extension strength (Table 3).

Referring to the studies by Sato (Sato et al., 2009), Sonstroem and Morgan (Sonstroem & Morgan, 1989) and others, we selected items such as life satisfaction (LSI-A, with a full score of 20, hereinafter referred to as LSI), subjective well-being (PGC morale scale, with a full score of 23, hereinafter referred to as PGC), and activity of daily life (hereinafter referred to as ADL) for analyzing the effects of exercise intervention (Cicek et al., 2021). ADL is an archaic activity ability index with a total score of 13; scores of ≥10 denote an individual’s ability to roughly manage life, whereas scores of <10 are considered to denote an individual’s inability and the need for social and family help to complete self-care (Fadzil et al., 2022) (Table 3).

### 2.4. Intervention Content

The exercise intervention involved outdoor fitness walking (Murray et al., 1969). Each session comprised three parts: a 10-minute warm-up, 40-minute walking exercise (Van Uffelen et al., 2009), and 10-minute cool-down (Hogan & Santomier, 1984).

### 2.5. Analysis Method

**Figure 1:**
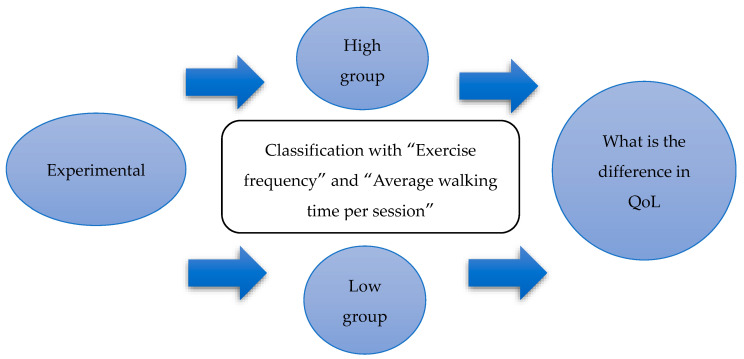
Research procedures.

The participants’ previous exercise habits were evaluated according to “Japanese exercise habits” outlined in the Sasakawa Foundation’s “Sports White Book” (Yu, 2020). Keywords used for post-experiment grouping were “Exercise frequency” and “Average walking time per session” (Figure 1).

Participants with an exercise frequency of “more than 2 times a week” and “less than 2 times a week” over the past year were categorized into the high- and the low-frequency exercise groups, respectively (Sato et al., 2009). Participants with exercise intensities of more than and less than 30 min per session were divided into the high and low average walking time per session groups, respectively (Mihalko, 1996).

Prior to the “Health Exercise Promotion Classroom” intervention, 34 participants had not engaged in any daily physical activities in the past year (Renaud et al., 2010); 54 participants had been engaged in sports activities “more than twice a week,” while 78 did so “less than twice a week.” Moreover, 83 participants spent more than 30 min per sports activity session, whereas 49 participants spent less than 30 min (Table 4).

### 2.6. Statistical Processing

Data were analyzed using SPSS 22.0 to determine the effects of the exercise intervention. Mean values of the variables measured before and after the exercise intervention were compared using t-tests. Spearman’s correlation analyses were performed to examine correlations among the study variables.

## 3. Results

### 3.1. Comparison of Body and Physical Strength Before and After the Exercise Intervention

The survey results indicated statistically significant improvements in blood pressure, abdominal curl, standing on one foot, and lower limb extension force. After the exercise intervention, the participants’ physical strength was found to be higher than the general physical levels in Japan (Yu, 2020).

The primary intervention in this exercise class focused on “fitness walks” conducted once a week for 60 min per session. This underscored the effectiveness of “aerobic exercises primarily utilizing the lower limbs” in regulating body weight, body fat, blood pressure, and other related metrics in middle-aged and older adults (Villareal et al., 2012).

Regarding body strength, significant changes were observed in weight (t = 6.52, *p* < 0.01, d = 0.122, [95% CI, 0.061, 0.182]), body fat percentage (t = 2.23, *p* < 0.05, d = 0.066, [95% CI, 0.033, 0.099]), diastolic blood pressure (t = 5.97, *p* < 0.01, d = 0.357, [95% CI, 0.181, 0.533]), and systolic blood pressure (t = 3.36, *p* < 0.01, d = 0.242, [95% CI, 0.122, 0.362]).

In addition, significant differences were observed in physical strength variables, namely sit-ups (t = 4.54, *p* < 0.01, d = 0.247, [95% CI, 0.126, 0.368]), standing on one foot (t = 2.52, *p* < 0.01, d = 0.367, [95% CI, 0.187, 0.554]), and lower limb extension (t = 5.96, *p* < 0.01, d = 0.323, [95% CI, 0.164, 0.462]) (Table 5).

The control group showed no significant differences before and after (Table 5).

### 3.2. Comparison of QoL Indicators Before and After the Exercise Intervention

In this study, QoL was assessed in terms of three indicators, namely LSI, PGC, and ADL. All participants demonstrated significant differences in ADL (t = 2.21, *p* < 0.05, d = 0.178, [95% CI, 0.091, 0.267]) and PGC (t = 2.69, *p* < 0.01, d = 0.171, [95% CI, 0.086, 0.256]) (Table 6).

The control group showed no significant differences before and after (Table 6).

### 3.3. The Exercise Frequency and QoL

In terms of exercise frequency, the “More than twice a week” group demonstrated significant differences in ADL (t = 2.32, *p* < 0.05, d = 0.277, [95% CI, 0.141, 0.414]) and PGC (t = 2.77, *p* < 0.01, d = 0.257, [95% CI, 0.130, 0.384]); however, the “Less than twice a week” group showed no significant differences in any of the variables. After exercise intervention, the LSI mean increased in the “Less than twice a week” group but decreased in the “More than twice a week” group (Table 7).

These results indicate that an exercise frequency of more than twice a week positively influences activities of daily living and perceived general health in middle-aged and older adults, although its effect on the LSI remains nonsignificant. Conversely, an exercise frequency of less than twice a week leads to noticeable improvements only in life satisfaction (Yamauchi et al., 2005).

### 3.4. The Average Walking Time per Session and QoL

In terms of exercise intensity, the “More than 30 min” group demonstrated significant differences in ADL (t = 2.64, *p* < 0.01, d = 0.347, [95% CI, 0.177, 0.518]) and PGC (t = 2.36, *p* < 0.05, d = 0.193, [95% CI, 0.097, 0.289]) after the intervention. The “Less than 30 min” group showed significant differences in LSI (t = 2.14, *p* < 0.05, d = 0.221, [95% CI, 0.116, 0.326]). However, in terms of the mean LSI, the “Less than 30 min” group exhibited an increase after the exercise intervention, whereas the “More than 30 min” group showed a decrease (Table 7).

These results indicated that exercising for more than 30 min had a positive effect on activities of daily living and perceived general health in middle-aged and older adults; however, it did not significantly enhance the LSI. Conversely, exercising for less than 30 min could significantly improve life satisfaction among middle-aged and older adults (Peig-Chiello et al., 1998).

### 3.5. “Exercise Frequency,” “Average Walking Time per Session,” and QoL Parameters

Exercise frequency showed a significant positive correlation with both ADL (rs = 0.298, *p* < 0.01) and PGC (rs = 0.243, *p* < 0.05). The average walking time per session exhibited a significant positive correlation with ADL (rs = 0.224, *p* < 0.05) (Table 8).

## 4. Discussion

### 4.1. Effect of Exercise Intervention on Body and Physical Strength

Although weight, body fat percentage, diastolic pressure, systolic pressure, and other parameters showed statistically significant changes before and after exercise, the actual magnitude of change was small. Planned exercise can effectively help middle-aged and older adults lose weight and body fat as well as reduce blood pressure (especially diastolic pressure). While the reductions in body weight and body fat are statistically significant, the actual decreases are modest with a more pronounced effect observed in lowering blood pressure (particularly diastolic pressure).

### 4.2. Effect of Exercise Intervention on QoL Indicators

The results show that ADL and PGC improvements are statistically significant, but the actual magnitude of improvement is relatively small. This indicates that regular and planned exercise has positive effects on middle-aged and older adults; however, these effects may not be easily detected or felt in a short period. Therefore, a proper exercise plan and persistence are particularly important for maintaining health.

### 4.3. Relationship Between Exercise Frequency and QoL

The results showed that the ADL and PGC of middle-aged and older adults significantly improved with exercise performed more than twice a week, although the magnitude of improvement was small. These findings reflect the positive physical and psychological effects of regular exercise. We conclude that regular exercise (more than twice a week) has a beneficial impact on activities of daily living and subjective well-being in middle-aged and older adults. However, this study also supports the view that “moderate regular exercise is beneficial to health.” Excessively high exercise frequency may have potential negative effects on some aspects (such as life satisfaction), so individuals should reasonably adjust their exercise frequency according to their own circumstances.

The significant improvement in ADL and PGC in the “twice a week” group indicates that exercise intervention is effective for middle-aged and older adults who exercise more than twice a week. Although the magnitude of improvement is small, it significantly enhances their physical and mental functions (ADL) and psychological well-being (PGC). This aligns with the general understanding that exercise benefits health and emphasizes the importance of maintaining a certain exercise frequency (≥2 times/week).

### 4.4. Relationship Between the Average Walking Time per Session and QoL

Regarding exercise intensity, the group exercising for more than 30 min per session showed significant improvements in ADL and PGC, while the group exercising for less than 30 min showed significant improvement in life satisfaction (LSI). We can conclude that for the LSI indicator, shorter exercise duration (less than 30 min) is more effective, whereas a longer exercise duration (more than 30 min) may have a neutral or even mild negative impact on life satisfaction.

For middle-aged and older adults who aim to improve their physical function and emotional state, exercise interventions lasting more than 30 min have been shown to be a more effective strategy with more significant effects. However, for those whose main goal is to improve overall life satisfaction, exercise interventions of less than 30 min may be a better choice.

The results of this study show that this duration can bring significant improvement, but longer durations can actually lead to a decrease in this indicator. The impact of exercise duration on different psychological and social indicators in middle-aged and older adults is not always linear; especially for life satisfaction, there may be an “optimal duration” (less than 30 min), beyond which the benefits of exercise may decrease or even have slight negative effects. This may be related to fatigue, time pressure, or perceiving exercise as a burden, particularly among older adults who are just beginning to exercise or have weaker physical abilities.

### 4.5. Correlation Between “Exercise Frequency,” “Average Walking Time per Session,” and QoL Parameters

Exercise frequency is significantly positively correlated with ADL and LSI, indicating that the more exercise middle-aged and older adults engage in, the stronger their self-care ability tends to be and the higher their life satisfaction. There is also a significant positive correlation between exercise intensity and ADL, meaning that greater exercise intensity in older adults is associated with stronger self-care ability. Although this relationship is not very strong, it is statistically reliable.

This study found that older adults who exercise regularly and appropriately increase the intensity of their exercise benefit their self-care ability. Specifically, exercise can improve the daily self-care ability of middle-aged and older adults and enhance their life satisfaction. However, exercise is only one of several influencing factors, and its effect is not decisive. These results provide a scientific basis for encouraging middle-aged and older adults to engage in regular exercise in their daily lives.

## 5. Conclusions

This study focused on middle-aged and older adults aged 60 years and above who participated in the “Health Exercise Promotion Classroom” in Kagoshima, Japan. It primarily explored the relationship between participants’ exercise habits—specifically, exercise frequency and average walking time per session—and their QoL after a three-month exercise intervention. All participants exhibited improvements in various aspects of their QoL except for LSI. These findings are consistent with those of Kim and Hwang (2017), Sonstroem and Morgan (1989), and others, indicating that regular and consistent exercise positively affects the QoL of middle-aged and older adults (Villareal et al., 2012).

This study exemplifies the relationship between regular exercise and daily living (Nunley et al., 2000). The keywords “Exercise frequency” and “Average walking time per session” were used to categorize individuals into groups, which was followed by a comparative analysis. The groups engaged in high-frequency exercise and high average walking time per session exhibited improvements in subjective well-being, activity ability indicators, and other aspects. By contrast, the groups engaged in low-frequency exercise and low average walking time per session demonstrated improvement in the LSI. These results confirm that the degree of improvement following an exercise intervention differs among populations with varying exercise habits (Angevaren et al., 2008).

After the exercise intervention, the average values of participants’ physical and body strength measurements were found to be higher than the general physical levels in Japan (Lee et al., 2016).

Middle-aged and older adults who engaged in high-frequency exercise (more than twice a week) demonstrated improved activities of daily living and perceived general health; however, their life satisfaction index did not improve significantly. In other words, exercising more than twice a week may enhance the functional abilities and subjective well-being of middle-aged and older adults, but it does not appear to improve life satisfaction in this population. Conversely, exercising less than twice a week led to marked improvements in life satisfaction.

Older adults with an average walking time of more than 30 min per session showed improvements in activities of daily living and perceived general health but no significant changes in the life satisfaction index (LSI). In other words, walking for more than 30 min per session may benefit middle-aged and older adults in terms of functional abilities and subjective well-being but not in terms of life satisfaction. Conversely, those with a walking time of less than 30 min per session showed significant improvements in life satisfaction.

First, the improvements in subjective well-being and activity indicators among groups engaging in high-frequency and high-intensity exercise align with the findings of Kim and Hwang (2017) and Sonstroem and Morgan (1989), who reported that regular exercise fosters happiness and satisfaction in addition to enhancing physical fitness and endurance. Our results reaffirm that regular and structured exercise among middle-aged and older adults is closely related to their QoL.

Second, improvements were noted in the LSI among older adults engaging in low-frequency exercise and an average walking time of less than 30 min per session. This result suggests that the regular and structured activities implemented at the beginning of the experiment benefited even those who had never participated, or had participated less frequently, in sports during the past year in terms of LSI (Valentine et al., 2009).

The above explanation highlights the importance of establishing realistic and suitable exercise goals and plans tailored to individual circumstances, particularly for middle-aged and older adults.

This study examined the relationship between QoL and daily exercise habits through a 3-month exercise intervention. Except for LSI, the other two components showed significant and positive associations with QoL. The results of the grouping analysis based on exercise intensity and frequency indicated that an average walking time of more than 30 min per session is beneficial to ADL and PGC, whereas an average walking time of less than 30 min per session is beneficial only in terms of LSI. Accordingly, we conclude that under the same exercise intervention, the degree of improvement in QoL components varies depending on individual exercise habits.

The motivation and satisfaction levels of participants in the “Health Exercise Promotion Classroom” project vary. Similar to the interaction between regular exercise and self-worth proposed by Hogan and Santomier (1984), the habit of exercising regularly prior to joining a health program has a direct effect on physical fitness. Specifically, individuals engaging in high-frequency exercise aim to enhance their muscle endurance and physical strength through consistent activity. Conversely, those who do not exercise regularly or engage in low-frequency workouts tend to prioritize enriching their overall well-being. Therefore, providing appropriate psychological support and counseling regarding awareness and motivation, tailored to individual exercise habits, is essential before commencing physical education classes for middle-aged and older adults.

## 6. Suggestion

This study shows that the effectiveness of exercise interventions on the QoL of middle-aged and older adults under identical intervention conditions is closely related to their physical exercise habits (Hill et al., 1993).

The results of this study not only underscore the benefits of exercise for the older adult population but also emphasize the importance of regular and sustained exercise in this group. In the context of Japan and other countries with a continuously increasing aging population, this study provides a scientific basis for promoting exercise habits and improving QoL particularly among middle-aged and older adults.

This study draws on various experiences to enhance the application of its findings in future sports classroom activities. However, a more comprehensive analysis involving different intervention groups, a larger experimental sample size, and a more refined intervention design is needed in future research. Additionally, future studies should focus on formulating appropriate intervention cycles and durations to achieve more accurate and effective outcomes, ultimately benefiting sports programs for middle-aged and older adults in several Asian countries, including Japan.

In addition, the sample size (number of courses), funding, and article length restrictions limit the generalizability of the results. Future studies should incorporate a broader range of sports and a larger sample size to enable more targeted and comprehensive comparisons and analyses, thereby obtaining more detailed insights.

In the next study, we will further investigate the mechanisms and pathways through which exercise intervention affects the life satisfaction of middle-aged and older adults. We aim to explore how to optimize intervention design—such as implementing personalized programs, integrating cognitive–behavioral therapy, and enhancing social support—to improve both specific indicators and overall life satisfaction. In addition, we will conduct long-term follow-up to observe whether changes in life satisfaction are sustained with continued adherence to exercise.

## Figures and Tables

**Table 1:** Gender attributes.

Gender	n	%
Male	49	37.1
Female	83	62.9
Total	132	100

**Table 2:** Age attributes.

Age Groups (Years)	n	%
≥60	12	9.1
≥65	74	56
≥70	27	20.5
≥75	11	8.3
≥80	8	6.1
Total	132	100

**Table 3:** Investigation project.

Personal Attributes	Gender, Age
Physiologic measurements	Height, weight, body fat, blood pressure
Physical strength tests	Grip strength, sit-and-reach, sit-ups, single-leg standing, and lower limb extension
Sports habits	Exercise implementation in the past year, including exercise frequency and average walking time per session
Questionnaire survey	LSI-A, PGC, ADL

**Table 4:** Exercise implementation in the past year.

Frequency	n	%	Average Walking Time	n	%
More than twice a week	54	40.9	More than 30 min	83	62.9
Less than twice a week	78	59.1	Less than 30 min	49	37.1
Total	132	100		132	100

**Table 5:** Comparison of body and physical strength test before and after exercise intervention.

	Height (cm)	Weight (kg)	Body Fat Percentage (%)	Diastolic Pressure (mmHg)	Systolic Pressure (mmHg)	Grip Strength (kg)	Sit-and-Reach (cm)	Abdominal Curl (Number)	Standing on One Foot (Second)	Lower Limb Extension Force (kg)
Pre	155.85 ± 7.12	56.79 ± 8.38	32.27 ± 7.29	131.68 ± 16.15	75.89 ± 9.15	27.42 ± 7.22	38.18 ± 8.75	8.24 ± 6.18	86.02 ± 40.43	46.57 ± 18.18
post	155.89 ± 7.17	55.80 ± 8.12	31.78 ± 7.68	125.80 ± 16.87	73.68 ± 9.14	27.87 ± 6.41	38.66 ± 9.36	9.82 ± 6.63	94.53 ± 38.78	52.59 ± 19.15
t	1.12	6.52	2.23	5.97	3.36	1.58	1.12	4.54	2.52	5.96
*p*	n.s.	**	*	**	**	n.s.	n.s.	**	**	**
d	0.006	0.122	0.066	0.357	0.242	0.067	0.053	0.247	0.367	0.323
C-Pre	154.56 ± 6.45	57.49 ± 7.52	32.15 ± 6.43	130.85 ± 15.56	75.78 ± 8.78	27.58 ± 6.95	38.16 ± 7.14	8.12 ± 5.27	85.13 ± 38.46	47.61 ± 16.47
C-Post	154.48 ± 6.21	57.86 ± 6.74	32.25 ± 6.24	131.86 ± 16.47	74.62 ± 9.27	27.47 ± 6.76	38.12 ± 8.01	8.06 ± 5.48	85.18 ± 39.67	47.42 ± 18.65
t	1.31	1.06	1.22	1.15	1.46	1.53	1.67	1.38	1.24	1.17
*p*	n.s.	n.s.	n.s.	n.s.	n.s.	n.s.	n.s.	n.s.	n.s.	n.s.
d	0.013	0.052	0.016	0.063	0.128	0.017	0.005	0.011	0.002	0.012

* *p* < 0.05, ** *p* < 0.01, n.s.: not significant.

**Table 6:** QoL comparison before and after exercise intervention.

	ADL	PGC	LSI
Pre	12.52 ± 1.03	12.66 ± 3.16	10.61 ± 4.36
Post	12.68 ± 0.78	13.16 ± 2.74	10.73 ± 4.19
t	2.21	2.69	0.34
*p*	*	**	n.s.
d	0.178	0.171	0.029
Control group Pre	12.45 ± 1.11	12.87 ± 3.09	10.58 ± 4.26
Control group Post	12.38 ± 0.65	12.76 ± 2.97	10.57 ± 3.95
t	1.45	1.16	1.25
*p*	n.s.	n.s.	n.s.
d	0.076	0.036	0.002

* *p* < 0.05, ** *p* < 0.01, n.s.: not significant.

**Table 7:** Comparison of QoL parameters before and after the exercise intervention by frequency and intensity.

	More than Twice a Week Group (n = 54)			Less than Twice a Week Group (n = 78)			More than 30 min Group (n = 83)			Less than 30 min Group (n = 49)		
	ADL	PGC	LSI	ADL	PGC	LSI	ADL	PGC	LSI	ADL	PGC	LSI
Pre	12.44 ± 1.02	12.64 ± 3.32	10.93 ± 4.62	12.55 ± 1.11	12.78 ± 3.12	10.38 ± 4.24	12.48 ± 0.91	13.02 ± 2.82	11.08 ± 4.18	12.55 ± 1.22	12.24 ± 3.54	9.96 ± 4.58
post	12.69 ± 0.78	13.44 ± 2.92	10.55 ± 4.48	12.70 ± 0.82	13.01 ± 2.66	10.68 ± 3.87	12.76 ± 0.63	13.54 ± 2.55	10.56 ± 4.17	12.66 ± 0.98	12.70 ± 2.96	10.88 ± 4.20
t	2.32	2.77	0.79	1.32	1.19	1.02	2.64	2.36	1.48	0.58	1.48	2.14
*p*	*	**	n.s.	n.s.	n.s.	n.s.	**	*	n.s.	n.s.	n.s.	*
d	0.277	0.257	0.084	0.149	0.079	0.074	0.347	0.193	0.125	0.099	0.142	0.221

* *p* < 0.05, ** *p* < 0.01, n.s.: not significant.

**Table 8:** Relationship between exercise frequency, average walking time per session, and quality of life parameters.

	Number	Exercise Frequency		Average Walking Time per Session	
		Correlation Coefficient		Correlation Coefficient	
ADL	132	0.298	**	0.224	*
PGC	132	0.243	*	0.198	n.s.
LSI	132	0.142	n.s.	0.096	n.s.

* *p* < 0.05, ** *p* < 0.01, n.s.: not significant.

## Data Availability

All relevant data in this study can be obtained from the first author on request.
